# Clinical, Microbiological, and Biochemical Impact of the Surgical Treatment of Peri-Implantitis—A Prospective Case Series

**DOI:** 10.3390/jcm11164699

**Published:** 2022-08-11

**Authors:** Fernando Luengo, Myroslav Solonko, Javier Sanz-Esporrín, Ignacio Sanz-Sánchez, David Herrera, Mariano Sanz

**Affiliations:** 1 ETEP (Etiology and Therapy of Periodontal and Peri-implant Diseases) Research Group, University Complutense, 28040 Madrid, Spain; 2 Section of Periodontology, Faculty of Odontology, University Complutense, 28040 Madrid, Spain

**Keywords:** peri-implantitis, surgical, microbiology, surface decontamination, glycine

## Abstract

*Background*: The aim of this study, a prospective case series, was to evaluate the clinical, microbiological, and biochemical impact of the surgical treatment of peri-implantitis. *Methods*: Thirty subjects with diagnosis of peri-implantitis were treated following a surgical protocol including access flaps, surface decontamination with ultrasonics and glycine powder air-polishing, and systemic antibiotics. Disease resolution was defined by the composite outcome including presence of probing depths (PD) ≤5 mm, absence of bleeding on probing (BoP)/suppuration, and no additional radiographic bone loss (>1 mm). Regression analysis was used to evaluate the patient-, implant-, and prosthetic-related factors possibly influencing treatment outcomes. *Results*: Patients were evaluated at 6 months post treatment, demonstrating statistically significant reductions in PD (2.14 ± 1.07 mm) and increase in mucosal recession (1.0 ± 0.77 mm). Plaque, BoP, and suppuration were also reduced by 40.56%, 62.22%, and 7.78%, respectively. Disease resolution was achieved in 56.67% of patients. No significant changes were detected in microbiological parameters except for a significant reduction in proportions of *Parvimonas micra*. Similarly, the levels of the biomarker interleukin-8 in crevicular fluid were significantly lower at 6 months. *Conclusions*: The proposed surgical treatment of peri-implantitis demonstrated statistically significant clinical improvements although the impact on microbiological and biochemical parameters was scarce.

## 1. Introduction

Peri-implantitis is a bacterial biofilm-associated pathological condition affecting the tissues around dental implants and characterized by chronic inflammation in the peri-implant mucosa and subsequent progressive loss of supporting bone [1]. Although data on its prevalence are heterogeneous [2], ranging between 20 to 30% at patient level [3,4,5,6], it represents a relevant complication in current implant dentistry and has emerged as an important healthcare problem [7].

The peri-implant tissues affected by peri-implantitis exhibit clinical signs of inflammation, characterized by bleeding on probing (BoP) and/or suppuration, increased probing depths (PD), and/or mucosal margin recession in addition to radiographic bone loss compared to previous examinations [8]. Moreover, there is peri-implant progressive bone loss, which usually leads to exposure of the usually rough implant surface, leading to a more favorable environment for biofilm formation and more difficulty in its appropriate removal [9]. If this condition is untreated, the perpetuation of these inflammatory and tissue destruction conditions may lead to implant failure and loss [1,10,11,12]. Furthermore, the increased bacterial challenge and the subsequent chronic inflammatory reaction will result in an increase in the expression of inflammatory mediators, such as interleukin (IL)-1β, IL-6, IL-8, or tumor necrosis factor (TNF)-α, which, when present at high concentrations in the peri-implant crevicular fluid samples, have been correlated with peri-implant disease severity [13,14,15,16] and, conversely, its reduction with a successful peri-implantitis resolution [17].

Different protocols have been proposed in the treatment of peri-implantitis [18,19,20], but there is still no standard of care treatment [18,19,21]. This is probably due to the lack of agreed clinical or radiographic parameters to assess treatment outcomes. Although most studies have used reductions in the percentages of bleeding sites, reductions in probing depths, and stability of peri-implant bone levels, there is no clear agreement of their specific significance, and hence, the use of a composite outcome combining the presence on shallow PDs, no BoP, and suppuration (SUPP) as well as no additional bone loss has been recommended [22].

Non-surgical treatment approaches have shown heterogeneous and non-predictable results, usually characterized by reductions in the levels of bleeding and inflammation but without concomitant reductions in PD and increase in bone level [23,24,25,26]. Recent clinical studies using novel approaches of non-surgical peri-implantitis therapy and combining mechanical debridement with systemic antibiotics have shown promising results although still unconfirmed by well-designed randomized clinical trials [27,28,29]. Due to this, the surgical treatment of peri-implantitis is currently the standard of care since it allows for a complete access to the implant surface for thorough decontamination, which has resulted in more predictable results for reducing the inflammatory changes in the peri-implant tissues and for arresting the disease process [30,31,32,33]. In combination with the surgical access to the affected implant, different surface decontamination methods have been evaluated although none has shown clear advantage in the improvement of clinical outcomes [32,34,35,36,37]. In vitro studies, however, have shown that air-polishing systems as compared to curettes, ultrasound tips, and titanium brushes result in a higher capacity for biofilm removal and, at the same time, for preserving the integrity of the implant surface [38,39,40,41].

When evaluating the topical use of systemic antibiotics in the non-surgical treatment of peri-implantitis, the single application of minocycline microspheres led to significantly higher PD reduction but comparable BoP changes, while repeated applications yielded significantly greater BOP reduction but similar PD changes compared to the control sites [42]. There is only one RCT evaluating the efficacy of the topical application of minocycline ointment adjunctive to the surgical treatment of peri-implantitis that has reported significant clinical improvements in terms of greater mean PD reduction and radiographic marginal bone levels compared to the control implant sites, while changes in BOP/SUPP were comparable between test and control groups [43]. Similarly, RCTs have investigated the potential benefits of the administration of systemic antibiotics along with surgical treatment of periimplantitis, reporting a positive effect of systemic antibiotics on the success of treatment (i.e., PD ≤ 5 mm, no BoP/SUPP, bone loss ≤ 0.5 mm) during a 1-year period but only for implants with modified surface characteristics [44]. The benefits of the systemic antibiotic regimen, however, did not last through the 3-year follow-up, leading to similar changes in BoP, SUPP, PD, and RBL values [36].

Considering the lack of a clear superiority in the specific surgical design or decontamination method to treat peri-implantitis-affected implants, we have designed this prospective case series study to evaluate the performance of a treatment protocol that includes access flap surgery, implant surface decontamination with glycine powder air polishing, and post-surgical administration of systemic antibiotics. Treatment outcomes were evaluated using the usual clinical outcomes as well as the preferred composite outcome of disease resolution that combines clinical and radiographical parameters. Furthermore, the impact of the proposed treatment protocol on microbiological and biochemical biomarkers has been studied.

## 2. Materials and Methods

### 2.1. Study design

This prospective case series study was designed as a 6-month, one arm, longitudinal clinical study to evaluate the outcome of a proposed peri-implantitis treatment protocol using clinical, radiographic, microbiological, and biochemical parameters.

*Study population and ethical considerations:* Consecutive patients participating in the supportive periodontal/peri-implant care (SPIC) program at the Post-Graduate Periodontal Clinic at the Faculty of Odontology, Complutense University (Madrid, Spain), were recruited when at least one dental implant in their mouth was diagnosed with peri-implantitis. Selected patients were included in this investigation after being informed on the objectives and characteristics of the clinical study and after signing an informed consent previously approved by the Ethical Committee (San Carlos Hospital, Madrid, Spain; register number: C.I. 12/209). This study followed the ethical principles based on the Declaration of Helsinki, and its reporting in this manuscript follows the criteria of the STROBE guidelines.

The selection of the study patients was based on predetermined inclusion and exclusion criteria:

*Inclusion criteria:*

- Presence of at least one implant with peri-implantitis, defined as: radiographic evidence of bone loss >2 mm, inflammation of the peri-implant mucosa as defined by positive BoP and/or suppuration, and at least one site with PD ≥ 5 mm.
- Based on the radiographic examination, the affected implant should not have a vertical peri-implant defect. Positive selection was based on the presence of peri-implant lesions wider than 4 mm, with an angle greater than 35°.
- In patients with a history of periodontitis, periodontal therapy should have been provided at least 6 months prior to the initiation of the study.

*Exclusion criteria:*

- Presence of relevant medical conditions and/or systemic medications that would contraindicate the surgical procedure or modify the tissue response after therapy.
- Patients requiring antibiotic prophylaxis.
- Heavy smokers (>10 cigarettes/day).
- Pregnant or lactating women.

### 2.2. Interventions

*Non-surgical phase:* Once enrolled in the study, patients were provided with standardized oral hygiene instructions and a session of non-surgical periodontal and peri-implant supra- and sub-gingival instrumentation, aiming to control inflammation on both teeth and implants. This therapy was provided using an ultrasonic device (miniPiezon^®^, EMS, Nyon, Switzerland) with specific tips for teeth (Instrument PS, EMS, Nyon, Switzerland) and implants (Instrument PI, EMS, Nyon, Switzerland). Polishing was then carried out using a rubber cup with a low-abrasive paste (Denta-Flux, Madrid, Spain).

**Figure 1:**
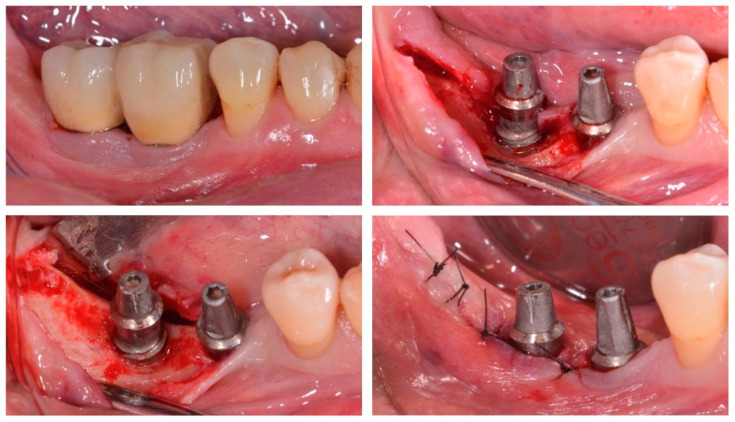
The surgical approach consisted of raising access flaps and implant surface decontamination.

*Surgical phase:* Three to four weeks later, the patients were re-evaluated, and if deep peri-implant probing depths with bleeding on probing remained, the surgical procedure consisting of the elevation of access flaps after intrasulcular and releasing incisions, for an adequate access to the treatment of the affected implant surface, was performed (Figure 1). After elimination of all the chronic inflammatory tissue, biofilm and calculus were removed from the implant surfaces using curettes and an ultrasonic device (miniPiezon^®^), with specific tips for implants (Instrument PS). The implant surface was thoroughly decontaminated (approximately 15 s at each site of the implant) by means of a glycine powder air-polishing system (Airflow Master Piezon^®^, EMS, Nyon, Switzerland). When needed, osteoplasty using carbide burs under profuse irrigation was used to favor flap adaptation and wound closure. Flaps were then replaced and sutured with 5/0 polyamid suture (Supramid^®^, Laboratorios Aragó, Barcelona, Spain).

Post-surgically, patients received a prescription for systemic antimicrobials, consisting of metronidazole, 250 mg (Flagyl^®^ 250, Sanofi, Barcelona, Spain), and amoxicillin, 500 mg (Amoxicilina^®^ 500, Normon, Madrid, Spain), three times per day for 7 days. Patients were also instructed to rinse twice daily with a 0.12% chlorhexidine and 0.05% cetylpyridinium chloride mouth rinse (PerioAid tratamiento^®^, Dentaid, Barcelona, Spain) and to modify oral hygiene procedures for the first four weeks. After this period, patients were instructed to discontinue chlorhexidine rinsing and to resume normal oral hygiene procedures, including interproximal cleaning. At one and three months after surgery, patients were examined, and if needed, supragingival debridement with a rubber cup and polishing paste was performed. The final evaluation was carried out 6 months postoperatively.

### 2.3. Outcome Variables

*Clinical variables:* The following clinical outcome variables were registered by the same experienced examiner at baseline and 6 months post-surgery with a manual periodontal probe (PCPUNC157, Hu-Friedy, Chicago, IL, USA): PD, recession of the mucosal margin relative to the margin of the restoration (REC); presence/absence of BoP, SUPP, and presence/absence of dental plaque (PlI).

*Radiological variables:* Intraoral radiographs of the peri-implantitis-affected implant were taken at baseline and 6 months post-surgery. Changes in interproximal bone level (distance from the prosthetic connection platform to the bottom of the intraosseous defect) were measured in the obtained digital images using an image-processing software (Adobe Photoshop CC 2019, Adobe Inc, San José, CA, USA).

*Biochemical biomarkers:* Gingival crevicular fluid (GCF) samples were taken in each included implant from the deepest PD site with positive BoP at baseline and 6 months post-surgery, always prior to microbiological sampling. The sampling method included isolation of the area with cotton rolls and gentle cleaning with air and a gauze to remove supragingival plaque deposits and potential saliva contamination. Then, a paper strip of standard length and height (Periopaper^®^, Oraflow, Hewlett, NY, USA) was inserted into the peri-implant pocket until mild resistance was felt and left in place for 30 s. The volume of GCF was measured from the Periopaper^®^ strips using the Periotron 8000^®^ device. Subsequently, the paper strips were inserted in micro-centrifuge plastic tubes and immediately stored at −80 °C until their biochemical analysis. This analysis was carried out using a Luminex System (Luminex^®^ 200, Luminex Corporation, Austin, TX, USA) to determine concentrations of the following biomarkers: IL-1β, IL-6, IL-8, and TNF-α.

*Microbiological variables:* Immediately after taking the samples for biochemical analysis, microbiological samples were taken from the same sites at baseline and 6 months after treatment, using two consecutive sterile medium paper-points (Maillefer, Ballaigues, Switzerland). The sample technique consisted of isolation with cotton rolls to prevent saliva contamination, removal of all supragingival plaque with a gauze and application of compressed air, and insertion of two consecutive paper points that were kept in place for 10 s and then transferred into a screw-capped vial containing 1.5 mL of reduced transport fluid (RTF) [45]. Samples were transferred to the microbiological laboratory within 2 h.

At the laboratory, subgingival samples were homogenized by vortexing for 30 s and serially diluted in phosphate buffer saline (PBS). Then, 0.1 mL of each dilution was plated manually on the specific medium Dentaid-1 [46] for the detection of *Aggregatibacter actinomycetemcomitans* and incubated for 3 days in air with 5% CO_2_ at 37 °C. Samples were also plated into a non-selective blood agar plate (Blood Agar Base II^®^, Oxoid, Basingstoke, England) supplemented with hemin (5 mg/L), menadione (1 mg/L), and 5% sterile horse blood with 7–14 days of anaerobic incubation. Total anaerobic counts were calculated as well as counts of selected periodontal pathogens (*A. actinomycetemcomitans, Tannerella forsythia, Porphyromonas gingivalis*, *Prevotella intermedia*, *Parvimonas micra*, *Campylobacter rectus*, and *Fusobacterium nucleatum*). In addition, the frequency of detection and the proportions for each bacterial species were also calculated.

### 2.4. Data Analysis

Although the primary outcome was the changes in PD, a composite outcome of disease resolution was also used to assess the performance of the treatment protocol [22]. Secondary outcome variables included other clinical parameters such as REC, BoP, PlI, SUPP, radiographic bone levels, biochemical concentration of the selected biomarkers and presence and frequency of detection, and proportions and counts of putative periodontal pathogens. Although during the study, all implants presenting with peri-implantitis were treated, only one implant per patient was selected for the analysis. Implant choice was based on the one with the worst clinical condition.

Clinical, microbiological, and biochemical variables at the selected implant were calculated first by patient and then by visit. Data were expressed as means and standard deviations (SD) for quantitative outcomes and as percentages in the case of qualitative outcomes. Data were tested for normality by means of a Shapiro–Wilk test. To compare quantitative variables, paired *t*-tests were used when a normal distribution was confirmed, while alternatively, Wilcoxon rank sum test was selected for non-normal distributions. Qualitative variables were compared with the McNemar test.

For the outcome disease resolution, a regression analysis model was constructed to assess the effect of different patient-, implant-, and prosthetic-related factors. The statistical analysis was performed using SPSS software (SPSS^®^ 20.0, SPSS Inc., Chicago, IL, USA).

The level of statistical significance was set at 5% (*p* < 0.05).

## 3. Results

Thirty consecutive patients (12 males and 18 females) were enrolled in the study and attended all visits, as defined in the clinical protocol. Their mean age was 62.3 years, and 37% were smokers of less than 10 cigarettes per day, 10% were former smokers, and 53% were non-smokers. Five of the patients were totally edentulous (17%) with complete implant-supported prostheses. All implants were located at the premolar or molar areas, with 53% in the maxilla and 47% in the mandible.

**Figure 2:**
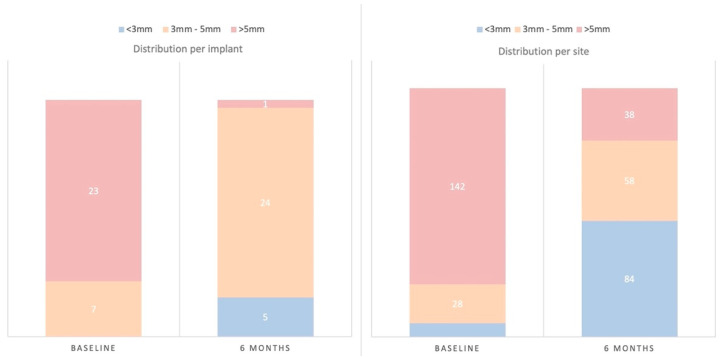
Distribution of implants and sites with probing depths (PD) <3 mm, between 3–5 mm and >5 mm at baseline and at the 6-month visit, expressed as percentages (%).

- *Clinical outcome variables:* PD showed, at 6 months post-surgery, a significant mean reduction of 2.14 mm (SD = 1.07) (*p* < 0.001), with a significant concomitant mean increase in REC of 1.00 mm (SD = 0.77) (*p* < 0.001) (Table 1). Implant sites with PD >5 mm at baseline showed PD of 3 mm or less, at 6 months post-surgery, in 76.67% of the cases; only one implant (3%) maintained sites >5 mm after therapy (Figure 2). Implant sites with PD between 3–5 mm at baseline were maintained within this category in 57.14% of the cases, while in 42.86%, it was reduced to <3 mm.

**Table 1:** Clinical variables, at baseline and 6-month visits, for probing depth, mucosal recession, and radiographic bone loss, expressed in mm (mean ± standard deviation (SD)), and bleeding on probing, suppuration, and plaque levels, expressed as percentage (mean ± SD).

	Probing Depth	Recession	Bleeding on Probing	Suppuration	Plaque	Radiographic Bone Loss
**Baseline**	5.80 ± 1.17	0.09 ± 0.17	90.00% ± 17.29	7.78% ± 18.94	72.22% ± 34.56	3.79 ± 1.15
**6 months**	3.66 ± 0.69	1.09 ± 0.74	27.78% ± 17.69	0.00% ± 0.00	31.67% ± 27.80	3.76 ± 1.27
**Change**	−2.14 ± 1.07	1.00 ± 0.77	−62.22% ± 24.34	−7.78% ± 18.94	−40.56% ± 26.51	−0.03 ± 0.61
***p*-value**	*p* < 0.001 *	*p* < 0.001 *	*p* < 0.001 *	*p* = 0.039 *	*p* < 0.001 *	*p* = 0.789

* Statistically significant changes (ANOVA) between baseline and 6 months (*p* < 0.05).

**Figure 3:**
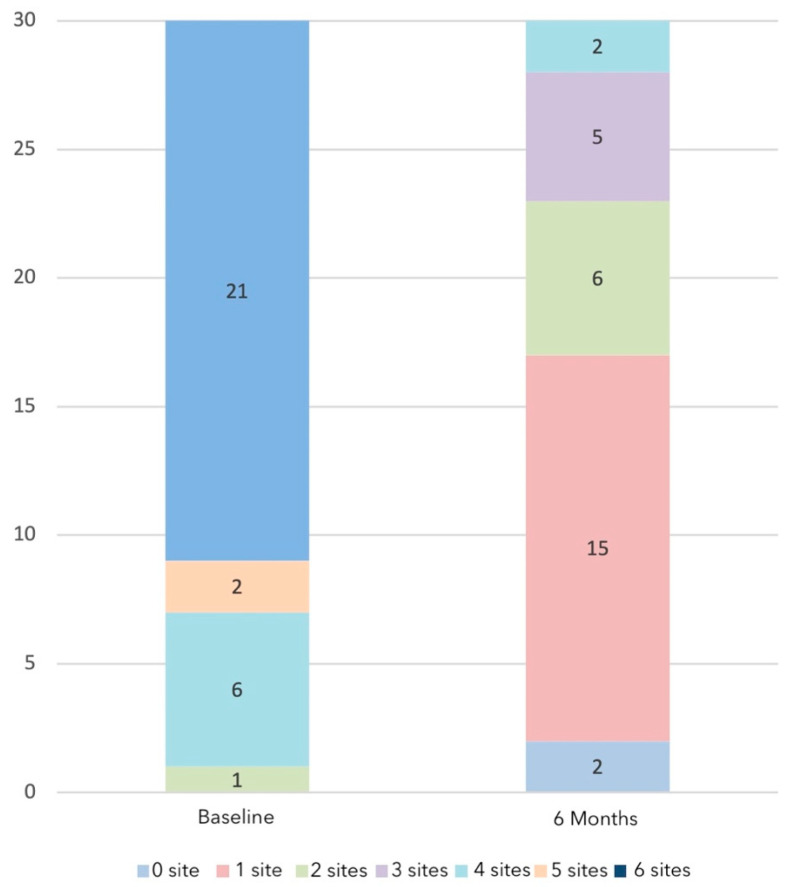
Distribution of the number of implant sites with bleeding on probing at baseline and 6 months.

BoP was present in 97% of the implants at baseline. After treatment, BoP demonstrated a significant mean reduction of 62.22% (SD = 24.34) (*p* < 0.001). Although reductions in BoP were statistically significant, and 56.67% of the implants only exhibited BoP at one site or less, only three implants had complete absence of bleeding (Figure 3). SUPP was not present at any implant at the 6-month post-surgery visit (*p* < 0.001). PlI showed a mean reduction of 40.56% (SD = 26.51) (*p* < 0.001). Radiographic analysis revealed a positive mean bone level change of 0.03 mm (SD = 0.61), and in only three patients (10%), an additional bone loss of 0.5 mm was detected.

**Figure 4:**
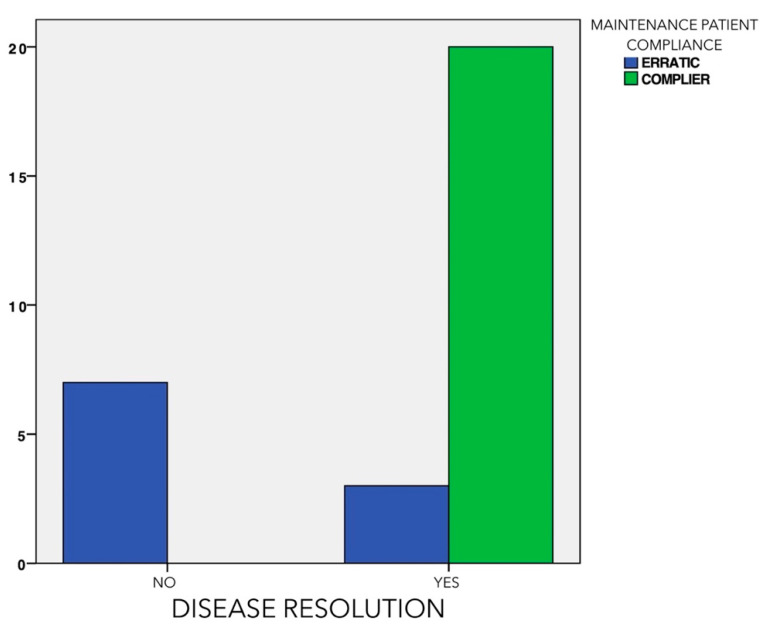
Association between the number of patients with compliance with supportive periodontal/peri-implant care visits and disease resolution at 6 months after treatment.

Disease resolution, defined with the composite variable PD <5 mm, absence of BoP (or in only one site around the implant), and no additional bone loss (<0.5 mm), was achieved in 56.67% of the implants (Table 2). For those implants not achieving disease resolution, different factors were identified as possibly influencing this treatment outcome (Table 3). Male gender (*p* = 0.015) and proper attendance to the SPIC protocol since implant placement (*p* < 0.01) were significantly associated with disease resolution. In fact, only 30% of the patients who did not properly attend SPIC had disease resolution after treatment, while all the patients that attended the prescribed sessions reached disease resolution (Figure 4).

- *Microbiological outcome variables:* Counts, proportions, and frequencies of detection of the targeted bacterial species did not show statistically significant changes (Table 4) except for the reduction in the proportions of *P. micra* (*p* = 0.023).
- *Changes in biomarkers in GCF:* The measured inflammatory biomarkers demonstrated reductions in their quantitative levels (expressed in pg.) although these changes were only statistically significant for IL-8 (*p* = 0.010) (Table 5).

## 4. Discussion

The present study evaluating a peri-implantitis treatment protocol combining surgical access flaps, implant surface debridement with air powder polishing, and systemic antimicrobials has shown a good therapeutic performance in arresting the peri-implant disease and in reducing the inflammatory condition of the peri-implant tissues, with a mean PD reduction of 2.14 mm, a mean BoP reduction of 62.22%, and complete elimination of suppuration.

Disease resolution was attained on 56.67% of the implants, and significant reductions were reported in peri-implant mucosal inflammation and in peri-implant probing depths. These results are consistent with previously published studies evaluating similar surgical protocols that have also reported percentages of disease resolution ranging between 26%% [32] and 79% [33]. These studies have reported results at different postsurgical follow-ups, such as one study reporting 30.4% disease resolution at different times between 2–11 years [35] or another study with 57.4% at one year [44] or 58% of implants successfully treated (no PDs over 4 mm) [47]. The lack of a higher degree of disease resolution reported in our investigation or in these similar prospective studies may be due to different factors, mainly the initial severity of peri-implant bone loss, which has been shown that, when affecting more than 50% of the implant length, significantly reduces the chances of success [48]. In the present study, in implants with initial PD >5 mm, only in 9% of them the PDs were reduced to <3 mm. However, in implants with initial PD of 3–5 mm, PD less than 3 mm was attained in 43% of sites.

The results from the present study showed complete absence of SUPP after therapy and absence of bleeding in 56.67% of implants (no bleeding or in only one site per implant). Similar studies have reported similar outcomes with post-therapy bleeding rates ranging between 23.8% [49] to 52.9% at six months and 41.9% at one year [44]. However, only three implants had complete absence of bleeding at all sites (six per implant). This finding has also been observed in similar clinical studies reporting 64% of implants with one or no sites with BoP [50]. In fact, a recent systematic review evaluating the efficacy of open flap surgery for treating peri-implantitis-affected implants reported a mean weighted effect of 34.81% in BoP reduction [24]. It is, therefore, difficult to interpret the clinical significance of BoP in isolated sites at implants after therapy, when PDs and marginal bone levels remain stable. The radiographic analysis has shown stable marginal bone levels after peri-implantitis therapy, with a mean gain of 0.03 mm at 6 months. Other studies have shown similar bone-level gains, ranging between 0.21 mm [44] to 0.5 mm [32,51].

Several systematic reviews [23,24,25,26] have shown that non-surgical treatment of peri-implantitis offers unpredictable results mainly associated with reduced levels of peri-implant inflammation but without a real impact on disease resolution. This mode of therapy, however, is highly recommended before the surgical treatment and should only be performed if disease resolution has not been achieved [22]. The adjunctive use of systemic antimicrobials has been proposed in most non-surgical [27,28,29] and surgical protocols [33,36,52] in order to halt the progression of progressive bone loss. In addition, the adjunctive use of systemic antibiotics, by administering a combination of amoxicillin and metronidazole, was used in the present investigation since all affected implants had moderately rough surfaces, and there is evidence of a beneficial short-term effect [50]. This added value of adjunctive systemic antibiotics has also been demonstrated in periimplantitis regenerative interventions [53], with beneficial effects maintained up to 5 years postoperatively in patients with adequate supportive therapy [33,52]. There is only one randomized clinical trial demonstrating that this adjunctive application has a significant short-term advantage versus the placebo when used on moderately rough surface implants [36], and its real impact on the peri-implant microbiota is presently unknown [18,54,55,56].

When considering the type of surgical approach, different surgical techniques have been proposed and evaluated in the treatment of peri-implantitis (access flap, resective, or regenerative), with its choice mainly based on the morphology of the defect and the objectives of the treatment (aesthetically relevant versus posterior sites) [57]. In non-regenerable peri-implant defects, the resective approach has shown significant long-term improvements in probing depth reductions and maintenance of stable peri-implant bone levels, also allowing for a better access to for proper biofilm control and maintenance [58], but this approach inevitably results in adverse aesthetic effects due to soft tissue recession. For this reason, access surgery is preferrable for attaining adequate access to the implant surface for thorough decontamination without compromising postoperative aesthetics [33,35].

The evaluation of the factors associated with a positive treatment outcome in the present study has identified patient compliance with SPIC as a key factor associated with the resolution of peri-implantitis, which also coincides with similar prospective clinical studies [33,47,59,60]. In addition to compliance with maintenance, the role of the patient in maintaining low plaque scores has been clearly highlighted in peri-implantitis treatment success [61,62] although in many cases, the prosthetic design may influence the ability of the patient to reach for adequate plaque control, and this circumstance has been related to the presence of peri-implantitis [63]. Conversely, it has been shown that in these cases, the modification of the prosthetic profile to improve the access for cleaning significantly influences the treatment outcomes [64]. In the present study, no prosthetic-related factor was identified as a deleterious factor although excess cement [65,66] and transepithelial abutment characteristics [67,68,69] have been shown to perhaps indirectly influence the development of peri-implantitis. Additionally, in this investigation, patient-related factors such as smoking did not show a significant influence on the treatment outcomes. This fact has also been reported in similar studies [47,50] even though smoking is a periodontal risk factor [70,71] and significantly influences the results of periodontal treatment [72].

In the present study, microbiological and biochemical parameters were also evaluated. The lack of a significant impact on these microbiological outcomes should be interpreted with caution considering the microbial diagnosis method used (anaerobic culturing) and the likely influence of the adjunctive systemic antibiotic therapy used. Furthermore, it is still controversial whether periodontitis and peri-implantitis share a similar pathogenic microbiota. In some studies, peri-implantitis has been associated with the well-characterized Gram-negative anaerobic microbiota with predominant periodontal pathogens (*T. forsythia*, *P. gingivalis*, *P. micra*, and *T. denticola*), while in others, there were distinct differences in the predominant microbiota [73]. Furthermore, we measured biomarkers associated with the inflammatory response and evaluated their impact after the treatment protocol used. However, only the levels of IL-1β and IL-8 were high at baseline and were indeed reduced after therapy, mainly IL-8. However, the levels of IL-6 at baseline were low, and TNF-α was almost undetectable. These results are different from other studies evaluating GCF of peri-implantitis sites, where levels of both IL-6 and TNF-α were high in peri-implantitis sites [15,74,75]. Nevertheless, there are studies [76,77] in agreement with our observations, with low or undetectable levels of TNF-α and no correlation with PD. Although concentrations of inflammatory biomarkers have been proposed as possible diagnostic tools for identifying peri-implantitis sites or sites associated with progression of this disease, there is not yet a valid predictive model using these biomarkers either to assess disease progression or treatment outcomes [13,78].

This prospective case series study has important limitations, mainly the absence of a control group, which precludes the evaluation of efficacy, and only allowing for assessing the performance of the proposed treatment protocol for peri-implantitis management. Moreover, both sample size and length of follow-up may be considered as limited. However, as a hypothesis-generating study, this clinical investigation provides important insight on the relevance of combining the surgical accesses to allow for proper implant surface decontamination.

## 5. Conclusions

In conclusion, and considering the reported limitations, the evaluated treatment protocol of peri-implantitis by combining the elevation of access flaps with implant surface decontamination, with air polishing with glycine, and systemic antibiotics including amoxicillin and metronidazole resulted in significant improvements of all evaluated clinical outcomes with a limited impact, however, on the measured microbiological or biochemical variables.

## Figures and Tables

**Table 2:** Components and combinations of the disease resolution outcome at the 6-month evaluation, expressed as number of implants (n) and percentage (%).

Component	n (%)
Probing depth (PD) < 5 mm	29 (97%)
No bleeding on probing (BoP)	17 (56.67%)
No additional bone loss (<0.5 mm change)	27 (90.0%)
**Combinations**	**n (%)**
PD < 5 mm and no BoP	17 (56.67%)
PD < 5 mm and no bone loss (<0.5 mm)	26 (86.67%)
No BoP and no bone loss (<0.5 mm)	17 (56.67%)
PD < 5 mm, no BoP, no bone loss (<0.5 mm)	17 (56.67%)

**Table 3:** Evaluation of different patient-, implant-, and prosthetic-related factors and disease resolution, expressed as absolute number of cases (n) out of total cases (N) and percentage of cases (%) where disease resolution was achieved after treatment in each category.

	Patient-Related Factors											
	Gender		Smoker			Additional Implants with Peri-implantitis				Compliance with Supportive Care		
	Male	Female	Non-smoker	Smoker	Former Smoker	No		Yes		Full Compliance	Occasionally/Not Attended	
**n/N (%)**	12/12 (100%)	11/18 (61.1%)	13/16 (81.3%)	7/11 (63.6%)	3/3 (100%)	10/12 (83.3%)		13/18 (72.2%)		20/20 (100%)	3/10 (30%)	
***p***	0.014 *		0.342			0.481				<0.001 *		
	**Implant-related Factors**											
	Platform switching connection		Mechanized collar neck		Presence of microthread		Implant design			Connection type		
	No	Yes	No	Yes	No	Yes	1-piece implant	2-piece implant		External connection	Internal connection	
**n/N (%)**	18/24 (75.0%)	5/6 (83.3%)	17/22 (77.3%)	6/8 (75.0%)	19/24 (79.2%)	4/6 (66.7%)	3/3 (100%)	20/27 (74.1%)		11/14 (78.6%)	12/16 (75.0%)	
***p***	0.666		0.896		0.517		0.314			0.818		
	**Prosthetic-related Factors**											
	Restoration size			Prosthesis material		Implant connection misfit		Prosthesis retention		Transepithelial abutment height		
	Single crown	Bridge	Overdenture	Metal–ceramic	Metal–resin	No	Yes	Screw-retained	Cement-retained	1 mm	2 mm	3 mm
**n/N (%)**	3/4 (75.0%)	16/21 (76.2%)	4/5 (80.0%)	19/25 (76.0%)	4/5 (80.0%)	12/16 (75.0%)	11/14 (78.6%)	20/24 (83.3%)	3/6 (50.0%)	4/5 (80.0%)	2/2 (100%)	½ (50%)
***p***	0.980			0.847		0.818		0.084		0.697		

* Statistically significant differences (chi-square test, *p* < 0.05).

**Table 4:** Mean counts and proportions (expressed as mean ± standard deviation) and frequency of detection of target bacterial species, with the number of positive samples at baseline and at 6 months.

	Outcome	*Total Anaerobic Counts*	*Aggregatibacter* *Actinomycetemcomitans*	*Porphyromonas* *Gingivalis*	*Prevotella* *Intermedia*	*Tannerella* *Forsythia*	*Parvimonas* *Micra*	*Fusobacterium* *Nucleatum*	*Campylobacter* *Rectus*
**Baseline**	Counts	6.14 ± 0.92	0.31 ± 1.17	3.62 ± 2.72	2.76 ± 2.46	1.12 ± 2.08	1.90 ± 2.41	2.80 ± 2.24	0.29 ± 1.12
	Proportions		0.06% ± 0.25	10.63% ± 15.97	3.40% ± 6.89	0.46% ± 0.95	5.14% ± 10.79	1.36% ± 2.10	0.14% ± 0.59
	Frequency		6.70%	66.70%	60.00%	23.30%	40.00%	63.30%	6.70%
	Detection (n)		2	20	18	7	12	19	2
**6 Months**	Counts	6.15 ± 0.76	0.14 ± 0.75	3.17 ± 2.76	2.31 ± 2.31	0.99 ± 2.02	0.92 ± 1.89	3.67 ± 1.82	0.23 ± 0.90
	Proportions		0.05% ± 0.29	12.37% ± 21.78	1.68% ± 4.25	0.46% ± 1.21	0.62% ± 1.32	2.22% ± 2.25	0.16% ± 0.83
	Frequency		3.30%	60.00%	53.30%	20.00%	20.00%	83.30%	6.70%
	Detection (n)		1	18	16	6	6	25	2
**Changes**	Counts	0.01 ± 1.13	−0.17 ± 0.83	−0.45 ± 3.31	−0.45 ± 3.24	−0.13 ± 2.67	−0.98 ± 2.92	0.86 ± 2.63	−0.06 ± 1.48
	*p*-value	0.766	0.180	0.475	0.449	0.929	0.088	0.084	0.715
	Proportions		−0.01% ± 0.32	1.74% ± 28.83	−1.72% ± 8.18	0.01% ± 1.49	4.52% ± 10.98	0.86% ± 2.87	0.02% ± 1.04
	*p*-value		0.655	0.732	0.317	0.656	0.023 *	0.088	1.000
	Frequency		−3.4%	−6.7%	−6.7%	−3.3%	−20.0%	20.00%	0.00%
	*p*-value		1.000	0.754	0.791	1.000	0.146	0.146	1.000

* Statistically significant changes (Wilcoxon test for comparison of proportions and counts; McNemar test for prevalence comparison) between baseline and 6 months (*p* < 0.05).

**Table 5:** Mean values (±standard deviation), expressed in pg, and changes in the different biomarkers, expressed in mean values and percentage (%).

	Interleukin 1β	Interleukin 6	Interleukin 8	Tumor Necrosis Factor α
**Baseline**	17.96 ± 30.98	0.70 ± 2.10	180.51 ± 240.99	0.65 ± 0.75
**6 months**	12.42 ± 30.10	0.52 ± 1.07	57.09 ± 51.70	0.49 ± 0.90
**Change** **(%)**	−5.54 ± 41.86 (−30.85%)	−0.18 ± 2.42 (−25.71%)	−123.42 ± 245.89 (−68.37%)	−0.16 ± 1.29 (−24.62%)
***p*-value**	0.474	0.692	0.010 *	0.490

* Statistically significant changes (*t*-test) between baseline and 6 months (*p* < 0.05).

## Data Availability

Not applicable.
